# Do We Need a Novel Framework for Classifying Psychopathology? A Discussion Paper

**DOI:** 10.32872/cpe.11699

**Published:** 2023-12-22

**Authors:** Winfried Rief, Stefan G. Hofmann, Max Berg, Miriam K. Forbes, Diego A. Pizzagalli, Johannes Zimmermann, Eiko Fried, Geoffrey M. Reed

**Affiliations:** 1 Clinical Psychology and Psychotherapy Group, Department of Psychology, Philipps-University of Marburg, Marburg, Germany; 2 Translational Clinical Psychology Group, Department of Psychology, Philipps-University of Marburg, Marburg, Germany; 3 School of Psychological Sciences, Australian Hearing Hub, Macquarie University Sydney, Sydney, Australia; 4 Department of Psychiatry, Center for Depression, Anxiety and Stress Research & McLean Imaging Center, McLean Hospital, Harvard Medical School, Belmont, MA, USA; 5 Department of Psychology, University of Kassel, Kassel, Germany; 6 Clinical Psychology Group, Department of Psychology, Leiden University, Leiden, The Netherlands; 7 Department of Psychiatry, Columbia University Vagelos College of Physicians and Surgeons, New York, NY, USA; Philipps-University of Marburg, Marburg, Germany

**Keywords:** ICD-11, DSM-5, Hierarchical Taxonomy of Psychopathology HiTOP, Research Domain Criteria RDoC, network theory, personality disorders, process-based therapy PBT

## Abstract

**Introduction:**

The ICD-11 and DSM-5 are the leading systems for the classification of mental disorders, and their relevance for clinical work and research, as well as their impact for policy making and legal questions, has increased considerably. In recent years, other frameworks have been proposed to supplement or even replace the ICD and the DSM, raising many questions regarding clinical utility, scientific relevance, and, at the core, how best to conceptualize mental disorders.

**Method:**

As examples of the new approaches that have emerged, here we introduce the Hierarchical Taxonomy of Psychopathology (HiTOP), the Research Domain Criteria (RDoC), systems and network approaches, process-based approaches, as well as a new approach to the classification of personality disorders.

**Results and Discussion:**

We highlight main distinctions between these classification frameworks, largely related to different priorities and goals, and discuss areas of overlap and potential compatibility. Synergies among these systems may provide promising new avenues for research and clinical practice.

## Highlights

- The World Health Organization’s International Classification of Diseases (ICD) and its latest revision ICD-11 offer the worldwide leading system for classification of mental disorders.
- Important proposals for rethinking classification came from the US National Institute of Mental Health’s Research Domain Criteria (RDoC), the Hierarchical Taxonomy of Psychopathology (HiTOP) initiative, and the systems/network approach to mental disorders.
- More ideographic approaches such as process-based interventions have also been suggested.
- We present different ideas for classification before we suggest ways in which these approaches can inform each other, while respecting the different purposes that motivated their development.

The classification of psychopathology has been a topic of debate for decades, sometimes from a scientific perspective, sometimes more from the perspective of societal relevance, epidemiology of clinical conditions, or in terms of its general usefulness. However, the discussion about how best to classify mental disorders has been particularly intense during recent years. These are not new discussion, but they were further stimulated by Insel’s assertion that the most widely used classification systems – the Diagnostic and Statistical Manual of Mental Disorders (DSM) and the International Classification of Diseases (ICD) – have not proven useful as a framework for research or in the development of new treatments targeted to underlying pathophysiological mechanisms (Insel et al., 2010). Since then, alternative approaches or extensions of these highly influential classification systems have been proposed and elaborated. Here, we will review various proposals for modifying the classification of mental disorders, including the most recent iteration of ICD’s chapter on mental, behavioral and neurodevelopmental disorders (ICD-11). We highlight similarities and differences between proposed alternatives and different frameworks of classification (i.e., RDoC; HiTOP; the revised classification of personality disorders, network approaches, process-based approaches), and explore their advantages and challenges.

The worldwide leading systems for the classification of mental disorders are the World Health Organization’s (WHO’s) ICD, currently in its Eleventh Revision (ICD-11) (WHO, 2022) and the American Psychiatric Association’s DSM, currently in its Fifth Edition (DSM-5-TR; American Psychiatric Association, 2022). Although they each have their own antecedents internationally, the ICD and the DSM have converged and diverged throughout their histories. The mental disorders chapter of the ICD-8 (WHO, 1967) and the DSM-II (American Psychiatric Association, 1968) were nearly identical and organized into the same three broad categories: psychoses; neuroses, personality disorders, and other nonpsychotic mental disorders; and mental retardation. Some of their basic concepts can be traced back to Pinel in 1798 (Postel & Quétel, 1994), Kraepelin (1893) and Bleuler (1911). The long history of concepts such as psychosis, schizophrenia, and depression could be attributed to the robustness of these concepts, or the resistance of the classification systems to change. The concepts were highly influential and the basis of research and treatment evaluations, but they were also misused (e.g., during mass murder campaigns like the “euthanasia program” in Nazi Germany). DSM-II integrated the numerical coding system of ICD-8. The descriptive, symptom-based approach that largely continues to characterize both the ICD-11 and the DSM was initially realized in the DSM-III (American Psychiatric Association, 1980), although there had been some parallel international developments. The DSM-III gained substantial international influence as a professional and commercial success, widely taken up by funders and researchers and selling a great many more copies than anticipated (Blashfield et al., 2014).

The ICD-11 classification of mental, behavioral and neurodevelopmental disorders and the DSM-5 were developed during overlapping periods of time and with substantial interaction between the WHO and the American Psychiatric Association. Intentional “harmonization” between the systems was most successful in terms of the overall organization of the classification, but the ICD and the DSM are currently more similar to one another than they have been in more than 40 years (for a detailed discussion see: First et al., 2021).

Most criticisms of categorical classification systems apply to both. These include questionable validity of many categories, dichotomization of dimensional features, high rate of use of “unspecified” or “other specified residual categories, lack of treatment specificity, excessive complexity and overspecification (Reed, 2010), reification (Hyman, 2010) (treating diagnostic categories as real and given without considering alternative approaches), heterogeneity of psychopathology / symptoms within diagnoses (e.g., Fried et al., 2016; Fried & Nesse, 2015; Hayes, Hofmann, & Ciarrochi, 2020), and stigma (Thornicroft et al., 2022), in addition to other issues that are explored in later sections of this article. Some aspects of the ICD-11 intended to address these issues and are explained in this article (e.g., secondary parenting, integration of dimensions, linkage to etiology, social and environmental determinants of health), and the solutions are based on the flexible digital infrastructure of the overall ICD-11 classification of diseases. At present, the ICD is more widely used in clinical systems around the world (Reed et al., 2011), whereas the DSM has been predominant in research.

The ICD and DSM classification systems are not intended or used for a single purpose (e.g., scientific validity), but rather have to achieve multiple goals at once. In part, they represent a pragmatic compromise among multiple competing demands and constituencies (Lilienfeld, 2014). From a clinical perspective, a key aim is to facilitate communication among clinicians and health system decision makers using the terms of the classification system. Diagnoses are meant to describe identifiable and meaningful clinical populations, a function that is intended to support treatment selection and clinical management. From a public health and policy perspective, an important priority is to communicate about the mental health of a population, and to quantify the need for treatment and governments responsibility to provide it. Economically, the definition of prevalence rates of specific syndromes and associated treatment costs, together with consideration of the disease burden and costs of untreated conditions, allow the proper allocation of limited financial resources. These policy and financial aims lead to highly influential decisions (e.g., allocation of financial budgets). And finally, the ICD must be acceptable and applicable all over the world to enable uniform global health statistics and to support comparability and focused prevention and intervention planning in support of global public health. The DSM, in contrast, is somewhat more bound to Western culture and in particular more influenced by the US legal and healthcare reimbursement systems.

Some experts now argue that the flaws inherent in these systems require a major shift in perspectives and principles for conceptualizing mental disorders. The Research Domain Criteria (RDoC) (Insel, 2014) advocates as a framework for research a focus on basic mechanisms of mental disorders that are based on scientifically well-defined psychological and neurobiological concepts. The Hierarchical Taxonomy of Psychopathology (HiTOP) (Kotov et al., 2017; Kotov et al., 2021) recommends using a more data-driven approach to define symptom clusters organised within broader dimensions. This approach has similarities to investigating the structure of personality traits, which resulted in the Big Five model (John et al., 2008). Meanwhile, these quantitatively based concepts enter more and more into the classification systems; the ICD-11’s classification of personality disorder and related traits (Swales, 2022; Tyrer et al., 2015) and the DSM-5’s Alternative Model for Personality Disorders (AMPD) (Zimmermann, Kerber, et al., 2019) are related examples.

Others reject these “nomothetic” classification approaches as they are predominantly oriented towards differences *between* persons, and instead highlight the importance of “ideographic” approaches studying processes *within* persons. This process-based approach not only advocates for a more individualized diagnostic process, but also a psychopathological understanding in the context of basic principles of evolutionary theory, focusing on aspects such as variation, selection and retention of psychological and social processes as typical and highly relevant adaptation strategies (Hayes, Hofmann, & Ciarrochi, 2020). Finally, and consistent with some of these frameworks, systems and network approaches view psychopathology as emerging from a complex system of biopsychosocial variables and processes (Borsboom, 2017; McNally, 2021), and treatment as effecting dynamic changes in these networks. Dynamic network theory not only considers the relation and centrality of symptoms, social and environmental influences and biological processes, but also the dynamics of change processes. This framework aspires to describe, understand, predict, and intervene on psychological processes of mental disorders, and also inspires work on bridging levels of analysis, such as connecting neurobiological to behavioral systems (Blanken et al., 2021).

These approaches raise important criticisms and offer important insights for potential paths forward, but the key question remains whether they are viable alternatives for meeting the uses and demands of existing classification systems, or parallel systems that can inform the ICD and the DSM. How can they be integrated with the knowledge that is in the DSM and ICD? Or are these recommendations for innovations just scientific “l’art pour l’art”, without relevant implications for clinicians or for public health?

In this article, we focus on these questions and hope to advance the scientific discussion concerning conceptualization of mental disorders, recognizing that the purposes of the ICD and DSM extend far beyond their use as a framework for research (International Advisory Group, 2011; WHO, 2019b). By bringing together authors working with very different theories and approaches, we introduce the background and rationales of these frameworks, starting with the reference system ICD-11 as a worldwide classification system with a long history and with important recent innovations in the classification of mental, behavioral, and neurodevelopmental disorders (Reed et al., 2022; Reed et al., 2019). We will investigate whether and how these new frameworks offer opportunities for improving the classification of mental and behavioral problems currently and over time.

## International Classification of Diseases, 11th Revision (ICD-11)

The WHO is a specialized, semi-autonomous agency of the United Nations with primary responsibility for global health. Its highest governance body is the World Health Assembly, which comprises the Ministers of Health of WHO’s 194 member states (countries). The WHO Constitution (WHO, 1948 reprinted in: WHO, 2020) provides a list of 22 specific responsibilities that were assigned to WHO at the time of its founding. Two of these are 1) to establish and revise as necessary international nomenclatures of diseases, of causes of death and of public health practices; and 2) to standardize diagnostic procedures as necessary.

The eleventh revision of the ICD, the ICD-11 (WHO, 2019a), was approved by the World Health Assembly on 27 May 2019 (WHO, 2019b). The ICD-11 represents the first major revision of the classification since the ICD-10 was published almost 30 years before (WHO, 1992) and incorporates major advances in research, practice, and information and healthcare technology. The primary purpose of the ICD is to serve as a framework for the collection and reporting of health information by its 194 member states. Important statistical uses of data based on the ICD include monitoring epidemics and other threats to public health, the calculation of disease burden, and the identification of vulnerable or at-risk populations.

After adoption of new versions of the ICD, the new system is implemented by member states as a part of their administrative, clinical, and information systems over the subsequent several years. Beyond meeting reporting requirements, many member states use the ICD as a part of the framework for defining their obligations to provide fee or subsidized health care to their populations. A specific consequence of this is that, in most countries, having a particular diagnosis generally entitles the individual to receive a specific range of health care services (e.g., a particular medication, a surgical intervention, a course of psychotherapy) that would not be provided without a qualifying diagnosis. In this way, the ICD is used by WHO member states as a framework for defining the universe of health conditions that are an appropriate basis for reimbursed health services by appropriately qualified professionals. Because of the ICD’s major implications for their health and health information and reporting systems, the pragmatic and statistical priorities of member states have a substantial influence on the ICD and its implementation. Member states are also invested in continuity across versions, so as not to undermine the usefulness of longitudinal health data.

The date of implementation of ICD-11 will vary by country, as it involves integration with laws, policies, health services and health data systems that vary considerably in scope and complexity. For example, the ICD-11 classification of mental disorders has been adopted clinically in Scottish mental health systems as of November 2022. Germany, on the other hand, intends to launch a fully integrated implementation covering both clinical and data systems in 2027.

### Development of the ICD-11 Classification of Mental Disorders

Although validity was obviously a primary concern in evaluating the need for changes in the mental disorders chapter of ICD-10 (First et al., 2015), developing the ICD-11 was not purely a matter of attempting to capture as well as possible the scientific “truth” about the nature of mental disorders (International Advisory Group, 2011). In developing the ICD-11 classification of mental disorders, the WHO Department of Mental Health and Substance Use also placed substantial emphasis on *clinical utility* and *global applicability*, which were seen as critical to the Department’s aim of reducing the global disease burden of these conditions (Reed et al., 2019). Detailed descriptions of different aspects of the development of the ICD-11 classification of mental disorders, its extensive program of integrated field studies, and its differences from the ICD-10 and from the DSM-5 have been provided elsewhere (First et al., 2021; First et al., 2015; Keeley et al., 2016; Reed et al., 2022; Reed et al., 2019).

In addition to the statistical version of the ICD-11 for Mortality and Morbidity Statistics (MMS) (WHO, 2023) the WHO Department of Mental Health and Substance Use developed Clinical Descriptions and Diagnostic Requirements (CDDR) for ICD-11 Mental, Behavioral and Neurodevelopmental Disorders. The CDDR are available on WHO’s ICD-11 website (https://icd.who.int/dev11/l-m/en) and will be published in book form in 2024. To enable mental health and other health professionals to understand and apply this part of the classification in their work with patients, the CDDR describe the features clinicians can reasonably expect to see in all cases of a given disorder and how to differentiate disorders from non-pathological expressions of human experience and from other disorders including medical conditions (First et al., 2015). The CDDR describes additional clinical features that can assist in evaluating diagnoses across cultures, genders, and the lifespan. (See First et al., 2015 for additional information about the contents of the CDDR and its development.)

### Benefits and Costs of Including Mental Disorders in the ICD

The ICD-6 (WHO, 1949) was the first version of the classification published by WHO, the first to include a classification of morbidity in addition to mortality, and the first to include a classification of mental disorders. (The ICD had previously been a classification of causes of death maintained by an international consortium. See Reed et al. (2016) for a historical perspective). The ICD-6 was therefore a major milestone in the recognition of mental disorders as valid health conditions and important causes of morbidity. In conceptualizing its approach to the development of the mental disorders classification in ICD-11, WHO’s International Advisory Group (2011) stated, the inclusion of mental and behavioral disorders alongside all other diagnostic entities in health care is an important feature of the ICD, facilitating the search for related mechanisms of etiology, pathophysiology, and comorbidity of disease processes and providing a solid basis for the parity of psychopathology with the rest of the medical system for clinical, administrative, and financial functions in health care” (p. 87).

At the same time, integration in the ICD has brought with it certain limitations because the ICD classification of mental disorders must follow the same structural and taxonomic rules as the rest of the classification of diseases. Clark et al. (2017) explain that the ICD-11 “remains structured as a categorical taxonomic system because this format is necessary for its application as the classification system for global health statistics and, to a large extent, for its use in clinical systems (e.g., in treatment selection and the determination of eligibility for health care services)” (p. 105). These requirements impose different and much stricter restrictions on the classification model than other models discussed in this article. Nonetheless, the ICD-11 has been able to introduce substantial innovations that move beyond a strictly categorical classification in in the direction of greater dimensionality, while at the same time respecting rules and conventions that have deep historical roots and are well accepted as the basis for classification in other areas of medicine.

The overall taxonomical rules inherent in the ICD — as a categorical classification system (and also inherent in the DSM, which in this regard is equivalent to the ICD) (Clark et al., 2017) — go back hundreds of years (Adriaens & De Block, 2013; Kendler, 2009) and have contributed to the reification or “essentialization” of mental disorder categories (Hyman, 2010). Specifically, it led to the illusion that ICD categories refer to discrete and non-overlapping disorders or subtypes of well-established validity, an illusion that has been further reinforced by the American Psychiatric Association’s focus on increasingly precise operationalizations of diagnostic criteria as a part of the DSM. Randomized controlled trials were based on these precisely defined patient populations, de-emphasizing areas of overlap and commonality that are highly relevant to real-world implementation (Tucker & Reed, 2008). Another limitation is that, by definition, a classification of diseases or health conditions locates the pathology within the individual.

### Moving Past Categorical Classification in the ICD-11

Structural and coding innovations introduced in the ICD-11, partly based on its fully electronic infrastructure, have made it possible to introduce classification innovations that expand beyond a strictly categorical approach to mental disorders. A core principle of taxonomic classification is that entities can be classified in one and only one place. ICD-11 uses a mechanism called “secondary parenting” to allow categories to appear in multiple places in order to improve clinical utility without sacrificing statistical integrity. For instance, Tourette syndrome is classified under movement disorders in the ICD-11 chapter on diseases of the nervous system but is also cross-listed under both neurodevelopmental disorders and obsessive-compulsive and related disorders in the chapter on mental, behavioral or neurodevelopmental disorders.

Moreover, the ICD-11 has made substantial progress in integrating a dimensional approach to the classification of mental disorders in the context of a categorical system (Bach et al., 2021; Clark et al., 2017; Gaebel, 2012; Reed, 2018). Classification entities were introduced that are not diagnoses on their own but can be appended to other diagnostic categories to characterize them by utilizing dimensional profiles. These include symptomatic manifestations of primary psychotic disorders (positive symptoms, negative symptoms, depressive mood symptoms, manic mood symptoms, psychomotor symptoms, and cognitive symptoms); prominent personality trait domains in personality disorders (negative affectivity, detachment, dissociality, disinhibition, and anankastia), and behavioral or psychological disturbances in dementia (psychotic symptoms, mood symptoms, anxiety symptoms, apathy, agitation or aggression, disinhibition, and wandering). Syndromal dementia diagnoses are rated for severity as well as these psychological and behavioral descriptors, and they are also linked to the presumptive underlying etiology (e.g., cerebrovascular disease, chronic use of alcohol, Parkinson disease, HIV). This provides a multidimensional picture of the individual clinical presentation.

### How Can Insights From Other Models Be Integrated – Incrementally – Into the ICD-11?

The ICD-11 is the first version of the classification that has been designed and built using a fully digital architecture. The coding system has changed from numeric (10 possible values per digit, i.e., 0 – 9) to alphanumeric (36 possible values per digit, i.e., 0 – 9 and A – Z), exponentially expanding the capacity of the system to contain information. Therefore, it is likely that the core architecture of the ICD-11 system will be in use for some time. Member states’ interests and priorities for health information are also unlikely to change dramatically in the immediate future. So, discarding the entire classification of mental disorders and substituting a fundamentally different approach will not realistically be possible anytime soon.

However, there is a well elaborated and already functioning system for making more incremental proposals for changes to the ICD-11 based on emerging evidence. Proposals can be made by anyone registered on the ICD-11 maintenance platform at https://icd.who.int/dev11/l-m/en. There are different proposal forms to modify the name or definition or other descriptive properties of a category, to add or delete a category, or to alter the organization of categories within or among groupings. After triage to verify that they meet basic requirements, proposals are sent to the Classification and Statistics Advisory Committee (CSAC), which primarily comprises representatives of the health statistics agencies of WHO member states. When appropriate, CSAC requests consultation from the Medical and Scientific Advisory Committee (MSAC) to evaluate the scientific and clinical foundation of a proposal and make a recommendation to CSAC on that basis. For MSAC, important factors in the evaluation of proposals are: 1) the amount and quality of scientific and clinical evidence in support of the proposal; 2) the amount and quality of contradictory evidence; and 3) the extent to which the proposal represents an international and widespread professional consensus. If a goal of the developers or adherents to any of the models discussed in this paper is to influence the ICD, the ICD-11 maintenance platform provides the best way to do that. The change in question should be proposed at a point where sufficient supportive evidence has been developed and there is substantial agreement (e.g., among international scientific and professional societies) about the desirability of adopting the proposal.

## A Paradigm Shift in Classifying Personality Disorders (PD)

A prime example of the advancement of ICD-11 is the section on PD. Research on PD has been at the forefront of challenging the validity of categorical classification systems in recent decades and has increasingly questioned their clinical utility (Bornstein & Natoli, 2019; Krueger, 2013; Widiger & Trull, 2007). For the ICD-11 PD Working Group, as well as for many other researchers in the field, the time was ripe for a radical change: developing a model that better represents the empirical evidence for the dimensional structure of PD (Hopwood et al., 2018). Although pragmatic concessions were made to certain stakeholders (e.g., by retaining the category of borderline PD), this goal was ultimately achieved (Tyrer et al., 2019). In this respect, the ICD-11 model for PD demonstrates that a paradigm shift within the established classification of mental disorders is indeed possible.

An important point of reference for the revision process was the AMPD, published in 2013 in DSM-5 Section III, which converges with some elements of the ICD-11 PD model. These include, for example, a refinement and substantiation of the general criteria for PD. Criterion A of the AMPD states that impairments in specific functions of the self (e.g., identity, self-worth, capacity for self-direction) and interpersonal relationships (e.g., capacity for empathy, cooperation, and intimacy) constitute PD and distinguish it from the state of mental health and other mental disorders. This definition is based primarily on the integration of various theories of PD (Livesley, 1998), but it is also compatible with the empirical finding that these features are particularly pure markers of the general factor of PD (e.g., Sharp et al., 2015). Furthermore, in the AMPD, the severity of PD takes centre stage and is directly represented diagnostically via a five-point rating scale—the Level of Personality Functioning Scale (LPFS: Zimmermann et al., 2023). The underlying evidence base included findings of the high predictive validity of severity with respect to future impairment, as well as its clinical usefulness in determining the amount of care required.

A particularly relevant element of the AMPD that converges with the ICD-11 PD model is using a dimensional trait model for describing the specific characteristics of PD. Here the goal was not to simply adopt an established model from personality psychology. The point was to adopt the predominant *methodological approach* of personality research by 1) aiming at efficient and precise description (rather than explanation), 2) collecting human judgments of hundreds of nuanced characteristics in thousands of self and other descriptions, and 3) conducting a comprehensive analysis of the covariation of those characteristics. Such a research program has contributed to a considerable integration of personality research since the 1990s. Most prominent examples are hierarchically structured personality models such as the Big Five (John et al., 2008) or HEXACO (Ashton & Lee, 2020), which encompass few broad domains and many specific, narrow facets. In line with this approach, the DSM-5 PD Working Group collected and defined 37 clinically relevant personality facets, created eight short descriptions per facet, submitted the entire list of items to multiple samples in self-report format, and used factor analytic methods to develop the taxonomy so that individual items are organized according to their empirical covariation (Krueger et al., 2012). The result is the AMPD trait model, with the five superordinate domains Negative Affectivity, Detachment, Antagonism, Disinhibition, and Psychoticism, and 25 subordinate facets. The fact that many domains correspond to the domains of the Big Five model (e.g., Negative Affectivity can be considered as the opposite pole of Emotional Stability) is ultimately an empirical outcome of this methodological approach and not an arbitrary decision by experts.

Some have called PD the “vanguard of the post-DSM-5.0 era” (Krueger, 2013). Indeed, the AMPD trait model has stimulated a large body of research over the past 10 years that tends to support its validity and clinical utility (Zimmermann, Kerber, et al., 2019, but also see: Clark & Watson, 2022), and the PD section in ICD-11 features for the first time a similar dimensional model in the main part of a classification system (Tyrer et al., 2019). Importantly, both models are based on a methodological approach that provides the template for creating a map for the totality of mental disorders, organized as they jointly emerge in the description of human raters. In this respect, the HiTOP initiative (Kotov et al., 2017) can be seen as an attempt to complete the work that has been started on revising the PD sections in DSM-5 and ICD-11.

## HiTOP for a Better Classification of Mental Disorders

**Figure 1:**
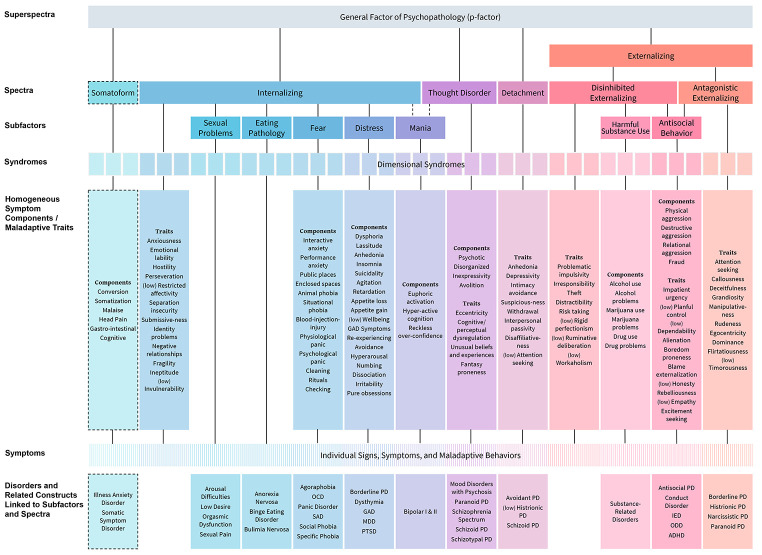
The Current Official HiTOP Framework *Note.* Dashed lines indicate dimensions included as provisional aspects of the framework. Abbreviations: ADHD, attention-deficit/hyperactivity disorder; GAD, generalized anxiety disorder; IED, intermittent explosive disorder; MDD, major depressive disorder; OCD, obsessive–compulsive disorder; ODD, oppositional defiant disorder; PD, personality disorder; PTSD, posttraumatic stress disorder; SAD, separation anxiety disorder. Reprinted from Forbes and Wright (2023), utilizing a Creative Commons 4 licence. See also HiTOP-system.org.

The Hierarchical Taxonomy of Psychopathology (HiTOP; Kotov et al., 2022; Kotov et al., 2017) represents a quantitative approach to the classification of psychopathology, extending the methodological approach of the AMPD described above. It is a hierarchical model of data-driven dimensions of psychopathology that have emerged in research on the structure of maladaptive personality as well as common and uncommon adult mental disorders (see Kotov et al., 2017 for the foundational review). The dimensions are based on patterns of co-occurrence or covariation among symptoms and disorders, and the hierarchy arranges these dimensions from individual signs and symptoms at the bottom all the way up to very broad dimensions at the top (e.g., a general factor of psychopathology, or p-factor; Caspi et al., 2014; Lahey et al., 2012). The model will be revised as the literature evolves and ultimately is intended to become a comprehensive framework articulating the empirical structure of all psychopathology (Forbes et al., 2023). The current model (Figure 1) is organised around six core *spectra* that largely mirror the personality domains described in the AMPD and the ICD-11 PD model. In this framework, diagnoses are not “present” or “absent”; individuals’ symptom profiles indicate severity to guide intervention at the level of components, syndromes, and/or spectra (Ruggero et al., 2019).

### Motives for HiTOP

The primary aim of HiTOP is to provide reliable and valid description of the structure of psychopathology to overcome the limited reliability and validity of many traditional categorical diagnostic categories (Kotov et al., 2017). HiTOP dimensions have already been found to outperform traditional diagnoses in predicting important outcomes in research and practice (e.g., impairment, treatment-seeking, and suicidality) (Kotov et al., 2021) and can be used for a variety of purposes — spanning understanding individuals’ symptom profiles, mapping the effect of a treatment to a specific domain of psychopathology, and quantifying risk factors that predict psychological ill-health and distress in the population (Conway et al., 2019). Further, the hierarchical nature of the framework provides a high degree of flexibility for researchers and clinicians to focus on the specific level of detail relevant to their research questions or clinical context without needing to compromise on breadth of assessment (Ruggero et al., 2019).

### Bridging the Gap Between Clinical Applications, Basic Psychology, Neuroscience, and Other Sciences

Due to its flexibility and breadth, HiTOP can act as a framework for disentangling the shared and unique features, processes, mechanisms, and causes of psychopathology for work spanning clinical practice, basic research, neuroscience, and other fields related to the study of mental disorders (Conway et al., 2023; Kotov et al., 2022; Kotov et al., 2021; Latzman et al., 2020; Perkins et al., 2020). Using reliable and empirically based constructs to operationalize psychopathology can offer a way forward that frees research in these fields from the limitations of traditional diagnostic categories and may present new opportunities for progress in understanding the mechanisms that underlie psychopathology, as well as for developing more effective treatments. While the official HiTOP measure is still in development (Simms et al., 2022), HiTOP constructs can be assessed using existing measures and analytic frameworks, reducing barriers to immediate implementation (e.g., Conway et al., 2019; Jonas et al., 2022).

### Potential for a World-Wide, Transcultural, and Culture-Sensitive Approach

An important limitation of the evidence base for the HiTOP framework is the predominance of studies in homogeneous white and Western samples. There have been several large cross-cultural studies as well as some work on multi-group invariance by race, ethnicity, gender, age, and sexual minority status in US samples (Rodriguez-Seijas et al., 2023). However, these studies have typically been limited to examining the internalizing and externalizing spectra. Ultimately, the goal will be to have a classification system that has utility and is robust across sociodemographic and cultural groups, while also sensitive to differences between these groups. With more comprehensive research in this area, meaningful differences between groups may well emerge such that a more nuanced framework will be required that goes beyond a single structure. This is ongoing work in both the Diversity, Equity, and Inclusion Workgroup and the Revisions Workgroup in the HiTOP Consortium (Forbes et al., 2023; Rodriguez-Seijas et al., 2023).

### Increasing the Acceptability and Utility of HiTOP in Practitioner Groups

Recent research shows mixed results regarding the acceptability and utility of HiTOP among practitioner groups; indeed, these were not the major goals for the development of HiTOP. For example, Balling et al. (2022) found that clinicians rated HiTOP as having better clinical utility than the DSM when applying both systems to a clinical vignette. Raskin et al. (2022) also found support from psychologists for alternatives to the DSM-5 in principle, but in practice they were unfamiliar with HiTOP.

There is substantial work underway to increase the acceptability and utility of the HiTOP framework for practitioner groups. For example, there is work documenting the mapping between HiTOP constructs and existing interventions (Mullins-Sweatt et al., 2020); transdiagnostic treatments can be selected to target a range of related symptoms (e.g., Selective Serotonin Reuptake Inhibitors / SSRI or the Unified Protocol to treat symptoms across the internalizing spectrum; Kotov et al., 2017) or targeted treatments can be used for narrow symptom domains (e.g., exposure therapy for phobic anxiety or sleep restriction for insomnia). A Digital Assessment and Tracker (HiTOP-DAT) has also been developed that assesses symptoms and traits across the framework as well as functional impairment (Jonas et al., 2022). It can be used for scoring clients’ symptom profiles at intake with reference to population norms, treatment planning, tracking progress over time, and cross-walking elevated HiTOP domains to ICD-10-CM codes for reimbursement and administrative purposes. Other clinical tools—such as links to existing ‘HiTOP Friendly Measures’ and explanations of how to use HiTOP in practice—are available on the HiTOP Clinical Network website (HiTOP-system.org; see also HiTOP Consortium, 2023) and field trials are underway at nine clinical sites to identify and address gaps in clinical utility (Kotov et al., 2022).

## RDoC for a Better Conceptualization of Mental Disorders

### Motives for RDoC

Launched in 2009 by the National Institute of Mental Health (NIMH) in the US, the Research Domain Criteria (RDoC) represents a research framework – rather than a nosological system – developed to overcome serious limitations associated with symptom-based diagnostic categories. Among others, three problems inherent in categorical classification systems (e.g. DSM, ICD) fuelled the development of RDoC (Insel et al., 2010). First was the fact that DSM/ICD diagnoses remain generally agnostic with respect to underlying pathophysiology and etiology. Second was the amply documented observation that current diagnoses are characterised by a remarkable degree of clinical (and presumably, etiological and pathophysiological) heterogeneity and extensive comorbidity. And finally, a substantial body of evidence indicates that DSM/ICD diagnoses are poor predictors of treatment response and clinical course.

The RDoC research framework responded to these challenges by focusing on functional dimensions divided into seven domains ranging from normal to abnormal. These dimensions include negative valence systems, positive valence systems, cognitive systems, systems for social processes, arousal/regulatory systems, and sensorimotor systems. The investigation of these dimensions occurs across seven units of analysis: genes ↔ molecules ↔ cells ↔ circuits ↔ physiology ↔ behavior ↔ self-reports. This approach fosters a multi-faceted assessment of mental disorders. Additionally, the framework acknowledges that both neurodevelopment and environmental influences continuously shape and affect the domains and units of analysis. For more information about RDoC, see reviews by Cuthbert (2020); Morris et al. (2022).

In contrast to descriptive approaches for the classification of psychopathology, RDoC was launched from the premise that disorder categories should better consider diagnosis-relevant mechanisms. The first incarnation of the RDoC framework relied on the assumption that mental disorders are brain disorders that originate from dysfunctional brain neural circuits (Insel et al., 2010). A key underlying assumption was that such circuit-level abnormalities could be addressed by therapeutic interventions.

One foundational tenet of the RDoC is that studying mental disorders from the perspective of dimensions of measurable behavior and related neurobiological mechanisms could overcome some limitations of current nosological systems (Cuthbert, 2022). Accordingly, this approach starts from basic knowledge about functions (e.g., ability to learn from rewards, propensity to attend to threat, working memory abilities), which can be evaluated at neural, behavioral, or self-report levels of analysis, for example. Within this conceptualization, mental disorders can be studied as disruptions in these functions resulting in abnormalities across levels of analyses (and with varying degrees of disruption) (Morris et al., 2022).

### Refinements, Misconceptions and Criticisms of RDoC

Partially due to early writings emphasising that mental disorders are fundamentally disorders of aberrant brain circuits (e.g., Insel et al., 2010), a misconception quickly arose that neural circuitry was considered the “primary focus” for RDoC (or stated differently, that neural units of analysis should be prioritized). This misconception has been clearly refuted in later writings (e.g., Kozak & Cuthbert, 2016), which have emphasized that no unit of analysis should have precedence or preferential consideration. With five of the seven units of analysis being biological, the RDoC retains a strong focus on biological mechanisms, but this should not be misconstrued as biological reductionism (since self-report and behavior are considered equally important). Rather, the RDoC framework emphasizes an approach in which mental disorders are studied simultaneously through observable (and quantifiable) behaviors as well as neurobiological variables.

Since its launch in 2009, the RDoC initiative has been criticized for several reasons, including insufficient attention to social determinants such as poverty, social inequality, and other environmental factors (e.g., Dean, 2019), particularly in earlier RDoC conceptualizations. Although an exhaustive discussion of such criticisms goes beyond the scope of the current review, a few selected key criticisms are discussed (Dean, 2019; Peterson, 2015; Ross & Margolis, 2019; Weinberger et al., 2015).

Perhaps among the most important criticisms, which goes to the core of the RDoC, is that serious mental disorders are not merely extreme forms of a dimensional continuum (Ross & Margolis, 2019; Weinberger et al., 2015), but rather *qualitatively* different states. Accordingly, serious mental disorders are thought to arise due to pathological processes that fundamentally disrupt normal neurobiological function (Ross & Margolis, 2019). Along similar lines, it has been argued that variables summarized in the RDoC matrix regulate normal brain function, rather than disease states. According to these views, RDoC’s top-down approach rooted in seven predefined domains of functioning holds little promise towards better treatments. Instead, critics advocate for a bottom-up “disease model,” which starts with identification of etiological factors (e.g., genetic variants), which in turn informs pathophysiological investigations and ultimately leads to a revised nosological system and targeted treatments. A second important criticism is that, owing to the fact that knowledge about the brain is still limited, the RDoC matrix focuses on well-established pathways and thus neglects emerging neurobiological targets discovered, for example, through recent GWAS studies of mental disorders (Ross & Margolis, 2019). As an example, Ross and Margolis (2019) highlighted that, as of Spring 2019, the RDoC matrix included 33 mentions of dopamine or serotonin, 36 mentions of GABA or glutamate, without any mention of molecules recently implicated in risk for major mental disorders. Although both criticisms are legitimate, it is important to emphasize that one important misconception is that the RDoC matrix is a fixed and prescriptive structure, focusing only on a subset of mental disorders. However, the RDoC leadership has been clear that the RDoC should be conceptualized as “a set of dynamic principles with which the field can build a cumulating knowledge base about psychopathology” (Cuthbert, 2020, p. 84). Thus, it is expected that the RDoC matrix will continue to evolve as knowledge is discovered and replicated.

To conclude, RDoC is an approach that bridges the gap between clinical applications, basic psychology, neuroscience, and other sciences. It has the potential of changing education and training programs for clinicians by moving the focus from diagnostic groups to mechanisms of change. However, at present, it has not yet developed to answer societal questions, health economic questions, or transcultural issues.

## A Systems Perspective on Mental Disorder Research and Practice

**Figure 2:**
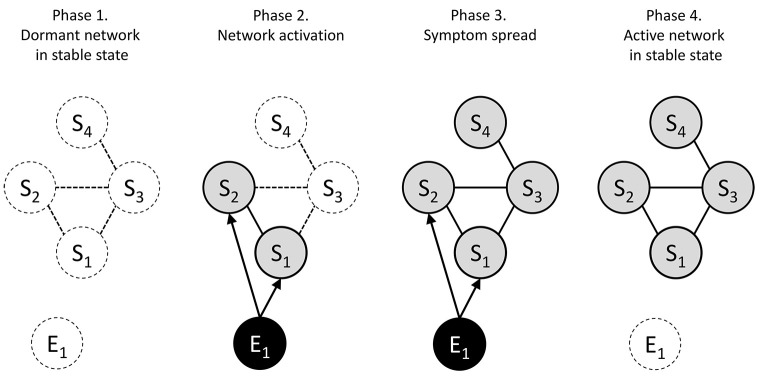
The Development of Mental Disorders From a Systems Perspective According to Borsboom (2017) *Note.* S = symptoms; E = environmental influences. According to the systems perspective, mental disorders go through several phases of development. Initially, there is an asymptomatic phase where the network is inactive (Phase 1). Then, an external event triggers some symptoms to manifest (Phase 2), which in turn enable other connected symptoms (Phase 3). If the network is highly interconnected, simply removing the external trigger does not result in recovery. This is because the network is self-sustaining and becomes trapped in an active, stable state (Phase 4). Figure reprinted with permission from Wiley & Sons Ltd.

Five key insights are of particular relevance to the systems perspective. First, mental disorders are highly multifactorial, including biological, mental, social, and environmental determinants. This contrasts with oversimplistic, monocausal frameworks that have dominated our field. Second, people with the same determinants can develop different problems (multifinality), and people with different determinants may develop the same problems (equifinality). This means it is difficult to predict how a person’s problems will develop over time. Third, people with the same diagnoses can differ substantially in both the determinants and problems they experience. There are (next to) no simple homogeneous categories, and one-size-fits-all treatments have shown limited efficacy. Fourth, the problems people experience are often causally related: for example, injury → pain → insomnia → decreased work performance → negative affect → relationship problems. Importantly, problems may persist even after determinants have subsided (see Figure 2). Overall, this calls into question simple cause-effect relationships as well as the clear separation of risk factors and symptoms. Fifth, mental disorders are dynamic: they rise and fall over time. Unfortunately, our knowledge of these dynamics is limited, largely owed to cross-sectional, between-subjects research designs.

### A Framework for Description, Prediction, Explanation, and Control

These five insights have led some experts to conclude that rather than studying single, isolated disorders or components, we should study the *systems* from which mental disorders arise. The systems perspective (or network approach) to mental disorders proposes just that: to conceptualize mental disorders as complex systems, and to study the systems (defined as components and relations among components) that give rise to mental disorders. The perspective has gained prominence in the last decade, and primers on the framework are available elsewhere (Borsboom, 2017; Fried, 2022; Olthof et al., 2023; Robinaugh et al., 2022; Roefs et al., 2022). This brief section serves as a summary of the core points and available resources. Broadly speaking, the perspective offers new theories and methods that aim to facilitate 1) description, 2) prediction, 3) explanation, and 4) control (i.e., prevention and intervention) of psychological systems.

#### Description

One of the first steps to gain a better understanding into complex systems is data description and visualization. Researchers in the last decade have implemented network methods from systems sciences that help psychologists estimate and visualize the relations between variables in datasets. Such methodological tools are available for cross-sectional data, panel-data (e.g., multi-wave epidemiological data), and time-series data (e.g., ecological momentary assessment data collected multiple times a day for several weeks using smartphones, or digital phenotype data collected using smartwatches or other wearable devices). A recent primer paper provides an overview of these methods and discusses challenges (Borsboom, Deserno, et al., 2021), which was followed by further discussion of methodological limitations (Borsboom et al., 2022; Neal et al., 2022). Importantly, some network methods allow one to distinguish processes that can only be identified at the individual level from those that generalize at the group-level (Beltz & Gates, 2017). These types of network models can start to bridge the gap between within-person and between-person perspectives, and highlight the importance of disentangling differences between and within persons.

#### Prediction

Recent work has suggested that studying the dynamic features of disorder systems over time may enable researchers to predict upcoming transitions into and out of mental disorders (Olthof et al., 2020; van de Leemput et al., 2014; Wichers et al., 2016). System features that are predictive of upcoming phase transitions are called early warning signals. Such signals have been widely and successfully studied in other literatures such as ecology, and one of the most commonly discussed early warning signals in the psychopathology literature is critical slowing down (van de Leemput et al., 2014; Wichers et al., 2016) – a feature that systems may exhibit before a phase transition occurs, such as from a healthy to a depressed state. Importantly, there is some evidence that critical slowing and other early warning signals can be detected some time before the symptoms of a person change, offering potentially novel opportunities for the prevention of mental disorders (Fried et al., 2023).

#### Control

Climate scientists conceptualize the global climate as a system, and variables of interest, such as the global temperature, emerge from interactions among system components. Climate scientists can simulate interventions on the system by implementing control mechanisms (such as reducing CO_2_ emissions) and studying the outcomes for global temperature. Similarly, conceptualizing mental disorders *as systems* and quantifying components as well as relationships among components may afford our field novel tools to study interventions. Researchers in this field recently developed a toolkit for system interventions by combining the two disciplines of network psychometrics and control theory — the former is concerned with the estimation of network estimation in psychological data, the latter with the question of how to optimally control systems to achieve desired outcomes, such as reducing global temperature or mental disorders (Henry et al., 2022).

#### Explanation

In the summer of 2022, there were considerable shortages of sparkling water in Italy, and media also reported a potential beer production shortage in Germany—both “because” of the Ukraine war. This is the result of causal processes in a system: war → increasing energy prices → decreased ammonia (fertilizer) production that is very energy intensive → decreased CO_2_ production that is a byproduct of ammonia production → CO_2_ shortage that affects production of sparkling water and beer. Understanding these causal pathways helps with predicting future states of the system, as well as thinking of potential control operations (e.g., subsidizing ammonia production or finding alternative sources of CO_2_). This also applies to psychological systems, where thorough descriptions of a system, along with theory building and testing, could help to properly map out components and relations within a system, and lead to a better understanding. Using a complex systems approach, Robinaugh and colleagues developed a theoretical model that aims to explain panic attacks and panic disorder (Robinaugh et al., 2022). This model specifies all relevant components and their relations in mathematical form, and the paper discusses in some detail the value of formalizing theories as systems (see also: Borsboom, van der Maas, et al., 2021; Haslbeck et al., 2022; Robinaugh et al., 2021).

## Process-Based Therapy as a New Conceptualization of Problems and Treatments on an Individual Level

### The Goal of a Process-Based Approach: The Individual Perspective

Process-based therapy is a new approach to psychopathology and treatment (Hayes, Hofmann, & Ciarrochi, 2020; Hofmann & Hayes, 2019; Hofmann et al., 2021). From a process-based perspective, perhaps the most problematic approach of contemporary psychiatry and psychology is to study phenomena on a between-person level (group level), rather than on a within-person level (individual level), leaving idiographic issues buried in statistical variation (Fisher et al., 2018). By studying psychological phenomena almost exclusively at a between-person level (e.g., diagnostic categories), we miss out on the meaningful individual processes that are the main focus in clinical practice and might lead us to the actual underlying processes of treatment change.

A related problem, specifically related to psychotherapy, is the contemporary approach of studying treatment processes with traditional mediation analyses based on a cross-sectional view of group data, which assumes that treatment change is nomothetic (Baron & Kenny, 1986). Again, this assumption makes findings difficult to apply to individuals and has little relevance to clinical practice. To model processes of change, clinically meaningful intervention research needs to focus on variables longitudinally, allowing them to vary between and within individuals. Furthermore, the impact of therapy cannot reasonably be reduced to just one or a few mediators and moderators, nor by assuming that these variables are independent or that they form simple unidirectional, linear relationships (Hofmann et al., 2020). The process-based perspective instead posits that change processes can more accurately be described as patterns of multiple inter-related variables forming dynamic complex networks over time, in individuals.

### The Process-Based Framework

For these reasons, Hofmann and colleagues have advocated for shifting towards process-based therapy, or PBT (e.g., Hayes & Hofmann, 2021; Hayes et al., 2019; Hofmann & Hayes, 2019) with the aim of discovering what change processes underlie psychopathology and its successful amelioration, and refining our understanding of these processes to facilitate treating individuals in a flexible, more precise way. In transitioning to a PBT framework, the focus in clinical psychology is shifting from determining "what treatments work?" to exploring "how treatments work and why." The goal of PBT is to gain a comprehensive understanding of two aspects: 1) identifying the essential biopsychosocial processes to target in an individual based on their specific goals and stage of intervention, and 2) determining the most effective methods for targeting these processes, utilizing functional analysis, complex network approaches, and identifying core change processes derived from evidence-based treatments (Hayes & Hofmann, 2018). PBT shares goals with classical functional analysis, including the consideration of context and the usefulness of specific behaviors. However, PBT encompasses a wider range of processes and is specifically designed to be applicable and beneficial for clinicians (Hayes et al., 2019).

PBT also highlights the importance of distinguishing between therapeutic procedures and processes. Therapeutic procedures refer to the specific techniques employed by a therapist with the aim of helping a patient to achieve their individual treatment goals (Hayes & Hofmann, 2018). Processes occur primarily within the client, but they also involve interactions between the client and therapist, the client and other individuals, and even within the therapist themselves. These processes encompass dynamic, theory-based, progressive, and multi-level changes. PBT necessitates a comprehensive theoretical framework to encompass specific evidence-based therapeutic models, and it has adopted an extended evolutionary model to fulfill that requirement (Hayes, Hofmann, & Wilson, 2020). PBT views psychopathology as maladaptations to a particular context. From an evolutionary perspective, these maladaptations stem from issues related to variation, selection, and/or retention of specific biopsychosocial dimensions within that context. Within PBT, this framework is referred to as the Extended Evolutionary Meta-Model (EEMM). The EEMM serves as a tool for researchers and clinicians to identify, study, categorize, and address the processes involved in psychopathology. We have extensively described the key aspects of the EEMM, including variation, selection, retention, and context, and have applied these concepts across various domains (Hayes, Hofmann, & Ciarrochi, 2020).

**Figure 3:**
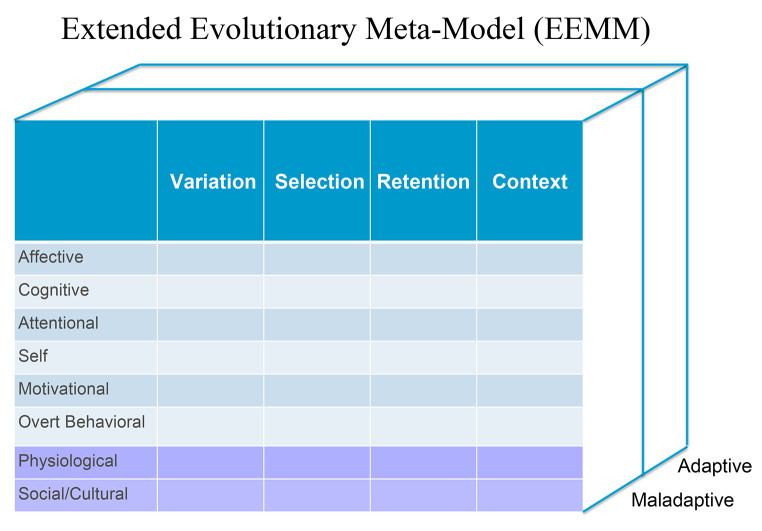
Extended Evolutionary Meta-Model of Change Processes *Note.* Figure from Hayes, Hofmann, and Ciarrochi (2020). For the meta-model, it was argued that variation, selection, retention, and context are constructs to explain whether adaptation processes to life challenges are successful or end up in psychopathological problems. The theory of evolution is used in all life sciences to explain complex living systems. It was argued by Hayes, Hofmann, and Wilson (2020) that evolutionary ideas have been underutilized by behavioral science. To introduce evolutionary thinking into the discourse, the extended evolutionary meta-model applies key concepts of variation, selection, and retention in different contexts to answer questions about the function, mechanisms, developmental pathways, and history of mental disorders. Six content dimensions, including affect, cognition, attention, motivation, self, and overt behavior, are discussed to specify adaptation processes, and to be essential for describing mental disorders. Figure reprinted with permission. Copyright S.C. Hayes and S.G. Hofmann.

*Variation* is the initial step toward adaptation (Hayes & Hofmann, 2018). It requires flexibility. Healthy selection is the second critical step in the process of adaptation. Even if there is healthy variation present, maladaptation can occur if beneficial psychological variants are not recognized and chosen. Selection processes include reinforcement, as well as the pursuit of goals, values, and attachment. Finally, retention involves intentionally developing and reinforcing adaptive patterns and habits to replace old maladaptive ones. Many evidence-based therapy techniques, such as homework assignments, aim to strengthen this aspect of adaptation. Often during the development of psychopathology, some behaviors and cognitive approaches tend to become habitual, resulting in a narrower range of variation. Thus, a dialectic relationship exists between variation and selective retention. Context serves as a moderating factor in this dialectic relationship, encompassing cultural, diversity, social support, and family factors. Psychological domains are not restricted to behaviors, but also include emotions, cognition, attention, self-perception, and motivational tendencies. Multilevel selection involves considering gene systems, behavioral classes, cognitive themes, physiological processes, and sociocultural influences. Together, these factors constitute the Extended Evolutionary Meta Model of change processes, as represented in Figure 3.

### Treatment and Research Implications of the PBT Approach

In recent years, there has been a growing trend towards transdiagnostic approaches in the field. The process-based approach addresses the limitations of the latent-disease model present in current classification systems by (1) systematically incorporating treatment processes from various therapy modalities and (2) viewing the treatment focus in PBT as the removal of unhelpful processes rather than a specified disorder. This approach has been developed to analyze individual-level change processes. PBT places emphasis on tracking the patient's progress over time, utilizing techniques such as ecological momentary assessment, wearables, and smartphones. By redefining symptoms as problems based on the patient's current experiences, the aim is to understand the processes that contribute to maintaining these problems and the functional relationship between them. Ultimately, the goal is to intervene effectively and predict future experiences. 

To understand the individual, PBT encourages us to study the individual in all its complexities. This approach may lead to exciting new avenues for psychotherapy research, both in terms of identifying processes with empirical support and new data analytic advancements (Hofmann et al., 2016). Further research is necessary to investigate whether the utilization of a PBT approach truly results in enhanced effectiveness of psychological treatment. This is because the scientific evaluation of this conceptual framework is still in its early stages, involving initial single case studies (Ong et al., 2022).

## Discussion

In this article, we have presented the different rationales and purposes of different approaches to the classification of mental disorders. After briefly summarizing these approaches, we will discuss how they can inform each other. It is clear that a major source of differences among the approaches presented relate to distinct goals and purposes. The primary aim of ICD-11 is as a tool for improving global public health, emphasizing usability and worldwide applicability. Given its foundational role in global health statistics, it has relevance for global development, economic evaluations, policy campaigns, legislation, and legal decisions. Currently, there is no real alternative that serves all these purposes. However, other approaches can stimulate changes and improvements that can either be integrated into the ICD-11 or can be further developed as a complementary or, perhaps eventually, alternative system.

HiTOP is an empirically-based proposal to organize symptoms according to a hierarchical and dimensional model. HiTOP has the potential to inform international classification systems because of its proximity to existing psychopathological concepts, but there is still a need for further evaluations based on HiTOP. The data underpinning the current HiTOP working model is heavily influenced by the traditional diagnostic categories it aims to improve, and the model does not yet capture sufficiently the diversity of populations. Further, previous research on HiTOP is largely focused on differences between persons, and such a nomothetic approach can suffer from limitations when being applied to individual cases (e.g., Fisher et al., 2018).

RDoC, at first glance, seems to be orthogonal to the classification approaches based on descriptive psychopathology. It follows the vision of identifiable, separable mechanisms that contribute to the development and maintenance of psychopathology. If such a system of identifiable mechanisms is further validated by empirical data, it can provide a breakthrough for moving primarily descriptive, psychopathological systems to a classification system that is characterized by central processes of mental disorders. However, many promises of RDoC have not been fulfilled yet. The definition of endophenotypes or the identification of central brain circuits responsible for mental disorders are progressing only slowly, and effect sizes of pharmacological treatments continue to be in a low to moderate range (Cipriani et al., 2018).

Another critique on RDoC is the tendency to focus on single systems, functions and mechanisms. Alternatives may include dynamic network models that take into consideration that relevant processes are interdependent. Dynamic network models can be applied to mental/psychopathological symptoms and processes as well as to neurobiological circuitries. Although not unique to network approaches, they allow for integration of machine learning techniques, for example to improve prediction of changes. However, so far, network approaches have been applied only in an initial series of studies. It remains to be seen whether this approach will lead to relevant new insights and to a profound change of our understanding of mental disorders.

The process-based approach mainly points to the fact that most diagnostic and interventional procedures focus on the individual, although the knowledge they are based on is mainly derived from analyses of group differences (the nomothetic-idiographic dilemma). The PBT approach advocates the need to collect more data on an individual level, such as individual trajectories about symptom development and recovery with the goal to derive novel, homogeneous, and treatment-relevant groups (using an integration of nomothetic and idiographic approaches such as the Group Iterative Multiple Model Estimation / GIMME algorithm; Gates and Molenaar, 2012). While the PBT approach offers a novel perspective on mental disorders and employs innovative analytical techniques, it currently lacks sufficient empirical validation.

### Opportunities and Barriers to Between-Framework Integration

HiTOP shares its methodological approach with the AMPD and ICD-11 trait models, resulting in high convergence between HiTOP spectra and extant trait domains (Wright & Simms, 2015). HiTOP is also similar to the ICD-11 in its focus on signs and symptoms and its prioritization of description as a foundation for explanation, and there is potential for more purposeful integration of HiTOP into ICD. One barrier will be the emphasis on pragmatism in the ICD-11 to ensure utility in health reporting and structuring clinical care, and also ICD’s worldwide perspective. Additional dimensions of psychopathology could be integrated into ICD-11 where sufficient evidence for higher order spectra, empirical syndromes and other dimensional constructs accrues.

Other dimensions or units of analysis such as those contemplated in RDoC (e.g., negative valence systems, arousal/regulatory systems, circuits) could also be incorporated into what is called the “foundation layer” of the ICD-11 without changing the statistical version. For example, the MSAC is already considering how best to incorporate genomic information in the foundation layer. Although this would not be a part of the statistical version, if specific genomic variables were already part of the foundation they could easily be moved into the statistical version as evidence accumulates and there is a strong clinical or public health rationale for doing that.

To take another example, the systems approach (e.g., Fried, 2022; Fried & Robinaugh, 2020) explicitly includes consideration of factors in the interpersonal, social, and physical environment, so a classification model that focuses solely on disturbances within the individual would initially appear to be a poor fit. However, the ICD-11 includes an extensive chapter on factors influencing health status and encounters with health services, which covers many of the important social and environmental determinants of health. These include finances, education, employment, drinking water and nutrition, social or cultural environment, and relationships, among other areas. Proposals based on the systems perspective could potentially focus on refining these categories and organizing them in configurations shown to be useful by research. The increasing attention currently being devoted to issues of health equity, with the goal of addressing the overwhelming evidence of serious and unequal problems with access to healthcare services, quality of care received, and unequal outcomes among minoritized groups across numerous health and psychological parameters (Kelly, 2022; WHO, 2018) suggests that consideration of these issues as part of the predominant global classification system for health could be important and timely.

Integration of HiTOP and RDoC is also a potential natural progression for both systems. For example, Michelini et al. (2021) worked on an interface linking RDoC and HiTOP dimensions to strengthen both systems: RDoC’s biobehavioral focus could improve research on the mechanisms and processes underpinning HiTOP constructs, and HiTOP constructs can be used as reliable phenotypes (clinical targets) to guide RDoC-informed studies. While reliable covariation does not necessarily indicate a shared cause among constructs, the flexibility of the HiTOP hierarchy can at least account for heterogeneity within traditional diagnostic categories and this integration of the two approaches offers a concrete path forward for determining whether and where biobehavioral mechanisms and processes map onto specific symptoms, broader components, or larger transdiagnostic dimensions (see also Tiego et al., 2023).

Despite the possibilities for integration between different frameworks, there are significant difficulties for integrating HiTOP and the systems perspective. One hurdle seems to be that the current HiTOP working model focuses on between-person differences, while the systems approach focusses primarily on within-person differences. However, it should be noted that HiTOP's underlying methodological approach of analyzing covariation in descriptions is also applicable to intensive longitudinal designs. In fact, similar to research on the Big Five as states (Borkenau & Ostendorf, 1998), the structure of within-person fluctuations in mental disorders is found to be largely compatible with the HiTOP spectra (Wright et al., 2023; Zimmermann, Woods, et al., 2019). In this respect, the HiTOP spectra could also have a heuristic value for the systems approach or PBT (e.g., regarding the selection of target dimensions or measures; Wright & Zimmermann, 2019). However, there are substantial philosophical and methodological differences between HiTOP and systems perspective frameworks. HiTOP – by design – searches for higher order latent factors of psychopathology while the systems perspective takes a deflationary stance on the existence of latent factors as constituents of mental disorders and instead emphasizes e.g., the importance of contextual variables for the development and maintenance of mental disorders (e.g., Borsboom, 2017). In systems approaches, mental disorders *are* a system of interacting problems *without simple underlying latent causes*. HiTOP, however, is hierarchical and models latent constructs (supraspectra, spectra and subfactors) with the use of dimension reducing techniques (e.g., Conway, Forbes, et al., 2022). Ongoing methodological and philosophical discussions (e.g., Borsboom et al., 2022; Forbes et al., 2021) exemplify the considerable challenge in integrating the systems perspective on mental disorders, including PBT, and HiTOP.

For RDoC, there is emerging evidence indicating that utilizing the framework in conjunction with categorical diagnoses, such as from DSM or ICD systems, may improve treatment outcomes. In a recent multi-site study in MDD, Ang et al. (2020) reported that behavioral (relatively better reward learning ability, as assessed by the Probabilistic Reward Task) and neural (relatively stronger resting state functional connectivity between the nucleus accumbens and the prefrontal cortex) reward-related markers predicted treatment response to the atypical antidepressant bupropion *after* failing 8 weeks treatment with the first-line treatment sertraline (an SSRI). Critically, without a priori incorporation of these measures (including of the RDoC subdomain of reward learning), identification of treatment-specific markers (moderators) of treatment response would not have been possible. This is consistent with the RDoC’s assumption that, by implementing quantifiable and granular assessments of fundamental dimensions of behavior that map onto precise neural circuitries (and computational parameters), we might be able to identify biologically more homogenous subgroups of individuals who might preferentially benefit from a given treatment strategy. For an important example of discovery of different “biotypes” in a study that used cognitive and electrophysiological variables to parse heterogeneity among a large group of individuals with schizophrenia, schizoaffective disorder, or psychotic bipolar disorder, see Clementz et al. (2016).

A systems perspective aims to identify shortcomings of traditional diagnoses, including inter-individual differences of people with the same diagnosis, lack of reliability and validity of categorical diagnoses, and an over-reliance on symptoms compared to other important factors. Generally, the systems perspective aligns well with PBT, given the explicit focus on studying networks of within-person processes. Methods from systems science can help to describe such systems, to describe their dynamic changes, and also to study to what degree systems generalize across people (Borsboom, Deserno, et al., 2021; Roefs et al., 2022). It also aligns with RDoC’s transdiagnostic focus on mechanisms, and much of the work done by RDoC can be framed as studying disorder / health components and their interrelationships.

The systems perspective and PBT also share a focus on understanding mental disorders as dynamic processes that are shaped by complex interactions among various factors (Borsboom, 2017; Hofmann et al., 2020; Hofmann & Hayes, 2019). Both approaches emphasize the role of individual experiences and the importance of context in shaping the development and maintenance of mental disorders. PBT and the systems perspective share a goal of developing personalized and context-aware treatment approaches that consider the unique needs and circumstances of the individual (e.g., Fried et al., 2023; Ong et al., 2022). Despite the similarities there are also differences between the approaches. PBT is primarily a treatment approach, while the systems perspective is a broader framework for understanding mental disorders. While PBT draws on the systems perspective to inform its understanding of mental disorders, it is primarily focused on developing and implementing novel interventions. The systems perspective, on the other hand, seeks to provide a comprehensive understanding of mental disorders that can inform the development of a wide range of future treatments.

### Conclusion

The field of diagnosis and classification of mental disorders is characterized by a rapidly developing discourse, the utilization of multiple novel frameworks, and efforts to effectively incorporate empirical data into the development of these models. As previously discussed, the main distinctions among the approaches result from their differing priorities and goals. However, many aspects of single frameworks can be integrated into one another, which could lead to promising new research programs and hopefully also spark ideas for effective psychological treatments along the way.
